# Cardiovascular Considerations of Experimental Hydroxychloroquine Therapy on Patients Diagnosed With COVID-19: A Case Series Review

**DOI:** 10.7759/cureus.9151

**Published:** 2020-07-12

**Authors:** Jay Patel, Radhika Patel, Lyd-Marie Rodriguez, Anamarys Blanco, Alan Hamza

**Affiliations:** 1 Internal Medicine, Ocala Regional Medical Center/University of Central Florida College of Medicine, Ocala, USA; 2 Family Medicine, Ocala Regional Medical Center/University of Central Florida College of Medicine, Ocala, USA

**Keywords:** covid 19, sars-cov-2, prolonged qt, bradycardia, risk-benefit

## Abstract

The severe acute respiratory syndrome coronavirus 2 (SARS-CoV-2) pandemic and its high virulence along with its variable presentation have generated a significant amount of interest within the medical community. The heterogeneous nature of the symptoms of the disease caused by SARS-CoV-2, coronavirus disease 2019 (COVID-19), ranging from being asymptomatic to severe acute respiratory distress syndrome (ARDS), has created significant interest in potential therapeutics. Given the lack of randomized controlled trials, most medications are experimental, and only anecdotal evidence is available so far regarding their efficacy. One medication that emerged as an early frontrunner as a promising therapeutic was hydroxychloroquine (HCQ), a common antimalarial and lupus drug. The adverse side effects that could result from its use did not gain much attention initially. We present the cases of two COVID-19-positive patients treated with HCQ at our institution, which showed adverse effects of the medication. While HCQ may have some therapeutic effect, it should be borne in mind that patients may experience more harm than benefit from its use.

## Introduction

The ongoing coronavirus disease 2019 (COVID-19) pandemic has led to global panic regarding its highly infectious process. In the race to find novel treatment strategies for COVID-19, numerous pharmacological agents are being touted as the silver bullet. The unfortunate reality of the COVID-19 pandemic is that, as providers, we are dealing with a highly virulent, variable, and potentially aggressive pathogen as evidenced by a reported R-naught (R0) of 5.7 [1]. Hydroxychloroquine (HCQ), a common antimalarial and lupus drug, has been shown to potentially reduce viral carriage and the number of symptomatic days in COVID-19 patients according to an open-label non-randomized French case study of 36 patients [2]. However, the findings of a subsequent randomized controlled trial have led to the FDA revoking HCQ's emergency use authorization [3]. The purpose of this case series was to highlight some of the cardiovascular complications related to HCQ and to engage in a risk-benefit analysis of its use in mild/moderate presentations of COVID-19.

## Case presentation

Case 1

**Figure 1:**
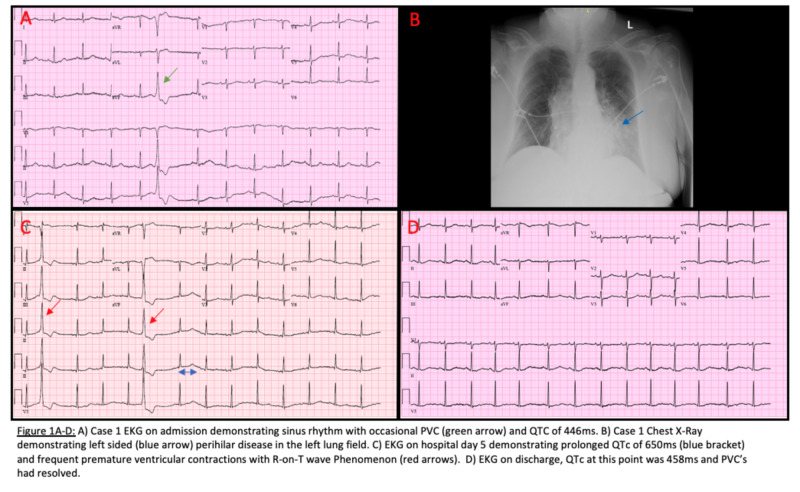
Case 1 examinations A) EKG on admission demonstrating sinus rhythm with occasional PVC (green arrow) and QTc of 446 ms. B) Chest X-Ray demonstrating left-sided (blue arrow) perihilar disease in the left lung field. C) EKG on hospital day five demonstrating prolonged QTc of 650 ms (blue double arrow) and frequent premature ventricular contractions with R-on-T wave phenomenon (red arrows). D) EKG on discharge; QTc at this point was 458 ms, and PVCs had resolved EKG: electrocardiogram; PVC: premature ventricular contraction

A 74-year-old female with a past medical history of coronary artery disease (CAD), chronic obstructive pulmonary disease, and autoimmune hepatitis on tacrolimus presented with a six-day history of fatigue, dry cough, shortness of breath, chest tightness, nausea, vomiting, and diarrhea. Her vitals were stable and the exam demonstrated bilateral decreased breath sounds and wheezing on admission. She was admitted initially for possible community-acquired pneumonia and started on empiric coverage of ceftriaxone and azithromycin (AZM). Ondansetron was also started due to her nausea. On day two of hospitalization, the patient's condition improved symptomatically, but she was subsequently found to be COVID-19- and influenza-B positive. HCQ and Tamiflu® (Roche Pharmaceuticals, Basel, Switzerland) were started while AZM was continued. Labs demonstrated troponin of 0.01 ng/ml (three sets), potassium (K) level of 3.2 mEq/L, magnesium (Mg) level of 1.8 mEq/L, and white blood cell (WBC) count of 3.4 thousand/mm^3^ at the time of initiation. On the morning of admission, the patient was found to have a QTc of 446 ms (Figure 1A). A chest X-ray demonstrated a left perihilar infiltrate (Figure 1B). Echocardiogram demonstrated left ventricular ejection fraction of 60% and no diastolic dysfunction. Clostridium difficile testing was negative. A repeat EKG on day five of hospitalization demonstrated prolongation of Qtc to 650 ms, and several premature ventricular contractions with R-on-T waves were noted (Figure 1C). She was asymptomatic at this time. HCQ, ondansetron, and AZM were subsequently discontinued. Furthermore, her electrolytes were optimized, specifically with Mg of >2.0 mEq/L and K of >4.0 mEq/L. By hospital day nine, the patient stated that her symptoms had improved. Her QTc was noted to be 458 ms on EKG on the same day (Figure 1D). She made an uneventful recovery and was discharged on hospital day 10.

Commentary

This patient had a moderate presentation of COVID-19 infection. Her case was complicated by co-infection with influenza B, radiographic evidence of perihilar infiltrate, and leukopenia. Given these combined findings, the initial benefit of HCQ seemed superior to the risk given the fear of rapid progression to severe acute respiratory distress syndrome (ARDS). However, after a day of supportive therapy, the patient's condition dramatically improved. It is important to note that her Tisdale score was 13 after HCQ, ondansetron, and AZM administration (high risk for QT prolongation) [4]. She was also having premature ventricular contractions (PVCs) throughout her hospitalization. Especially worrisome were R-on-T waves noted on her telemetry, which could be a forewarning for impending ventricular fibrillation, especially in the setting of acquired long QT and her history of CAD [5]. In this case, the patient’s hospital course was clearly complicated by HCQ in addition to the many QTc-prolonging agents she was prescribed; hence, the overall benefit seemed marginal.

Case 2

**Figure 2:**
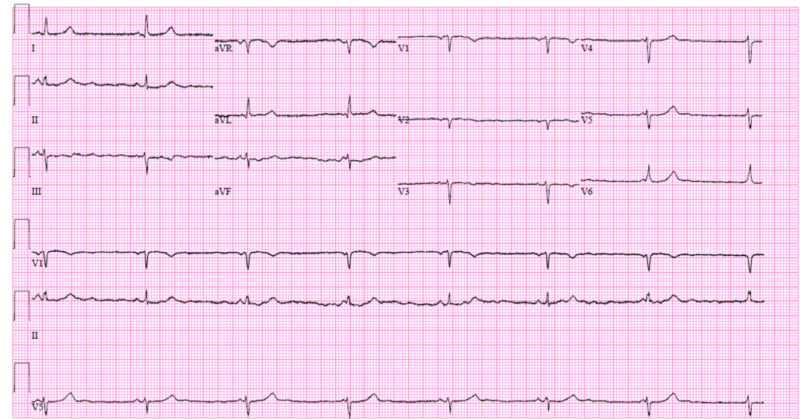
Case 2 EKG on admission demonstrating marked sinus bradycardia EKG: electrocardiogram

A 40-year-old female with no significant medical history presented with a one-day history of nausea, vomiting, and diarrhea. She stated that she had recently returned from Scotland and had begun experiencing symptoms of rhinorrhea starting in late January 2020. She was not tested immediately for COVID-19 due to a lack of fever. She was given a steroid dose-pack and a five-day course of AZM at that time. She improved initially but later relapsed one week prior to admission when she developed symptoms of shortness of breath and mild wheezing. After testing positive for COVID-19, two days prior to admission, her primary care physician started HCQ. While on this medication, she developed nausea, vomiting, and diarrhea within 48 hours and required admission for the administration of intravenous fluids. Her vitals demonstrated a temperature of 36.7 °C and a heart rate of 48 beats per minute (bpm). The exam demonstrated scattered bilateral wheezes and bradycardia. Lab values demonstrated point-of-care (POC) troponin of 0.02 ng/ml, brain natriuretic peptide (BNP) of 231 ng/L, K of 4.0 mEq/L, Mg of 1.8 mEq/L, and WBC of 17.3 thousand/mm^3^. Clostridium difficile testing was negative. Her EKG (Figure 2) noted marked sinus bradycardia with a ventricular rate of 44 bpm. The patient had no prior history of arrhythmias. It was believed that her symptoms of sudden diarrhea, nausea, and vomiting were secondary to HCQ as was the bradycardia. The medication was discontinued, and her symptoms resolved; her heart rate improved to 60 bpm, and she was uneventfully discharged on no antimicrobial medication.

Commentary

This case showed a mild incidence of COVID-19 in a patient who was at low risk for QT prolongation given a Tisdale score of 4. Given the low risk of adverse effects, it initially appeared reasonable that the patient’s primary care provider had started her on HCQ treatment. The marked sinus bradycardia was a worrisome finding that thankfully resolved with discontinuation of the medication. It should be noted that the patient’s baseline EKG was unknown. Providers should consider obtaining a baseline EKG, renal panel, and hepatic panel prior to the administration of HCQ in these patients. This case illustrates the fact that HCQ provided little benefit for such a mild presentation of COVID-19 as in this patient.

## Discussion

While HCQ was demonstrated to be effective in the French study by Gautret et al., it is not a proven treatment modality and it should be used with caution. The above two cases add to the ongoing discussion as to whether HCQ therapy in COVID-19 patients is beneficial for all, especially given how it was paired with another arrhythmogenic agent AZM. We believe these are among the first few cases illustrating adverse cardiovascular effects of the experimental five-day HCQ therapy in mild/moderate presentations of COVID-19.

While the safety profile on HCQ is relatively favorable, the drug is a well-known arrhythmogenic medication that can lead to life-threatening ventricular arrhythmias, most commonly recognized as Torsades de Pointes (TdP) [6]. Admittedly, the relationship between QTc prolongation and TdP is not linear; nevertheless, clinicians are well aware of the risks of prolonged QTc. The Tisdale risk stratification scoring system was invented for predicting the risk of QT prolongation [4]. As shown in Table 1 with respect to our patients, the Tisdale score characterizes patients as low risk (score<4), moderate risk (score 4-11), and high risk (score >11). Case 1, which was considered as high risk, involved a patient with potentially lethal asymptomatic prolongation of the QTc segment. Additionally, the R-on-T phenomenon noted on telemetry was worrisome as this could have potentiated a polymorphic ventricular tachycardia (PVT) or even ventricular fibrillation [5]. These were clear complications of the HCQ and, subsequently, prolonged the patient’s hospital stay. Case 2, considered as low risk, demonstrated how HCQ therapy initiated in an outpatient resulted in an adverse outcome that led to hospital admission. The bradycardia was thankfully recognized early as this could have progressed to potential atrioventricular block had the patient continued the medication [7]. Both of these cases throw light on HCQ-related complications resulting in prolonged hospital stay/hospitalization that exposed these patients to potential hospital-acquired infections or even re-infection with COVID-19, which has only been sporadically reported [8].

**Table 1:** Tisdale score for cases 1 and 2 Case 1 was classified as high risk (76%) for developing QTc prolongation. Case 2 was classified as low risk (15%) for developing QTc prolongation

	Points if Yes	Case 1	Case 2
Age ≥68 years	1	1	0
Female sex	1	1	1
Loop diuretic	1	0	0
Serum K+ <3.5 mEq/L	2	2	0
Admission QTc ≥450 ms	2	0	0
Acute MI	2	0	0
≥2 QTc-prolonging drugs	3	3	0
Sepsis	3	3	0
Heart failure	3	0	0
1 QTc-prolonging drug	3	3	3
Total		13	4

The American College of Cardiology (ACC) has issued guidance on how to proceed in patients who could potentially receive HCQ for COVID-19 therapy. In the outpatient setting, a baseline assessment with EKG, renal function panel, and hepatic function panel should be completed and, if possible, the EKG should be assessed by an electrophysiologist [9]. Furthermore, other QTc-prolonging agents should be discontinued if possible [9]. Relative contraindications in these patients would include: 1) history of long QT syndrome, 2) QTc of >480 ms, and 3) Tisdale score of ≥11 [9]. For inpatients, the recommendations include the same as above plus: 1) placing patients on telemetry 2) obtaining serum potassium on a daily basis, and 3) obtaining EKG two to three hours after the second dose of HCQ. Guidance on QTc increases of >60 ms or overall Qtc of >500 ms seems to point towards the discontinuation of AZM and subsequent dose decrease of HCQ. If QTc does not improve, the ACC recommends complete discontinuation of HCQ [9].

The emergence of new data regarding HCQ's application in the early disease course of COVID-19 has called its use into question. In the Outcomes Related to COVID-19 treated with Hydroxychloroquine among In-patients with symptomatic Disease (ORCHID) trial conducted by the Petal Network, 479 patients were enrolled in a double-blinded, placebo-controlled randomized trial to determine if five-day therapy with HCQ led to more favorable outcomes [10]. The dosing regimen included HCQ sulfate 400 mg PO twice daily on day one followed by 200 mg twice daily doses on days two to five [10]. The initial results from the trial suggested that there was no benefit or harm from HCQ use, and the trial was subsequently halted in mid-June 2020 [11]. The data is still awaiting full analysis for submission to peer review [11]. In June 2020, the FDA officially revoked emergency use authorization for HCQ as there was no benefit demonstrated from the aforementioned trial [3]. Moreover, the agency has cautioned against its use in the outpatient setting given its potential cardiovascular complications and inability to closely monitor patients [3]. The trial data and FDA recommendations in their totality demonstrate that HCQ use likely is not beneficial in the early disease course of these patients. Regarding the lack of harm shown by the ORCHID trial, more subgroup analysis is warranted in these patients to fully determine if patients with cardiovascular disease burden suffered harm, despite the absence of mortality among patients.

The potential for arrhythmogenic effects of these medications, especially in patients with cardiovascular disease, should be seriously weighed against their benefit before administration. Based on the recent preliminary analysis of data from the ORCHID trial, there is no benefit from HCQ use. If HCQ is commenced by providers for the treatment of COVID-19 in patients with a cardiac history, it should be done at the guidance of an infectious disease physician in conjunction with a cardiologist. While the prospect of potential lifesaving therapeutics seems tempting, the long-standing principle of “Do No Harm” is of importance now more than ever.

## Conclusions

Experimental therapy with HCQ in mild/moderate presentations of COVID-19 should be balanced with considerations of the risk of potential cardiac complications in both inpatient and outpatient settings. This is especially the case as there have been no double-blinded randomized control trials with results demonstrating that the benefits of the therapy outweigh the risks.
